# Effect of Seawater Curing Agent on the Flavor Profile of Dry-Cured Bacon Determined by Sensory Evaluation, Electronic Nose, and Fatty Composition Analysis

**DOI:** 10.3390/foods12101974

**Published:** 2023-05-12

**Authors:** Sol-Hee Lee, Hack-Youn Kim

**Affiliations:** Department of Animal Resources Science, Kongju National University, Yesan-Gun 32439, ChungNam-Do, Republic of Korea; chzh73@naver.com

**Keywords:** dry-cured bacon, natural curing agent, microbial composition, flavor, free fatty acid

## Abstract

The purpose of this study was to check the applicability of seawater as a natural curing agent by analyzing the difference it causes in the flavor of dry-aged bacon. Pork belly was cured for seven days, and dried and aged for twenty-one days. The curing methods included the following: wet curing with salt in water, dry curing with sea salt, brine curing with brine solution, and bittern curing with bittern solution. The seawater-treated groups showed a lower volatile basic nitrogen value than the sea-salt-treated groups (*p* < 0.05); dry curing showed a higher thiobarbituric acid reactive substance value than other treatments (*p* < 0.05). Methyl- and butane- volatile compounds and polyunsaturated fatty acids such as g-linolenic and eicosapentaenoic were the highest in the bittern-cured group, lending it superior results compared to those of the control and other treatments in sensory flavor analyses (cheesy and milky). Therefore, bittern is considered to have significant potential as a food-curing agent.

## 1. Introduction

Salt plays an important role in extending the shelf life of food and is especially essential in fermented foods that are manufactured through long-term curing and aging. If a large amount of salt is added to prolong the storage period, the water content decreases during the drying period and the salinity is relatively increased, which has a great influence on the taste. Salt affects the taste and aroma of fermented food because the water activity and level of microorganisms change depending on the amount of salt added and the salting method [1]. However, adding a lot of salt to food may lead to a propensity to develop coronary heart disease, hypertension, and stroke [2]. Therefore, it is necessary to reduce the amount of salt while maintaining the flavor, storage, and quality properties of salted foods.

Curing is largely divided into dry-curing and wet-curing methods; dry-curing methods are the most commonly used [3]. This is because the process is relatively simple, and aerobic and anaerobic microorganisms can directly decompose proteins and fats to express flavor. However, in the case of dry curing, a difference in salinity between the inner and outer membranes of the meat may occur [4]. Wet curing is divided into indirect curing for a certain time after putting the meat in the curing agent, and direct curing of the meat through injection [5]. In the former wet-curing method, the salt diluted in water easily penetrates the muscle due to osmotic dehydration, so it is easy to match the core and outer surface salinity [6]. Therefore, anaerobic microorganisms decompose proteins and fats during the wet-curing process; as a result, the resultant flavor from wet curing may be different than that from dry-curing methods.

Dry-cured meat products have been made up of several types of shapes, such as whole muscle meat products (*Jamón*, *Pancetta*, etc.) or ground meat products (*Salami*, *chorizo*, etc.). Depending on the size and shape of the protein and fat in the meat and on the curing method, drying speed and composition may differ [7]. Because of this, dry processing has various physical and chemical properties. In addition, dry processing improves the flavor of meat products by changing their physical and chemical properties [8]. The flavor of meat is generated by the breakdown of major precursors or thiamine, lipids, and small amounts of carbohydrates [9]. Fat is known to have a higher effect on the flavor of dry-cured meat products than protein, and the flavor derived from fat plays an important role in determining consumer preference [10].

Hundreds of volatile compounds develop during processing, and by identifying them, various actions can be predicted. Not all volatile compounds contribute to flavor; only aromatic compounds have a direct effect on flavor [11]. For flavor analysis, qualitative analysis methods such as the electronic nose (e-nose), and quantitative analysis methods such as gas chromatography–mass spectrometry (GC–MS) are used. The e-nose is used to roughly analyze and classify the aroma of food and determine the differences between samples [12,13], and the GC–MS enables quantitative and qualitative analyses of the flavor [14]. Principal component analysis and predicted volatile compounds analyzed by the e-nose can quickly and objectively distinguish the flavor of meat and are used in various fields of food for quality analysis/evaluation [15,16]. Many studies have reported that dry-aged meat has a stronger roasted and meaty flavor than wet-cured meat [17,18]. Therefore, a wet-curing + dry-aging solution that can enhance flavor substances holds good potential for new products.

**Figure 1:**
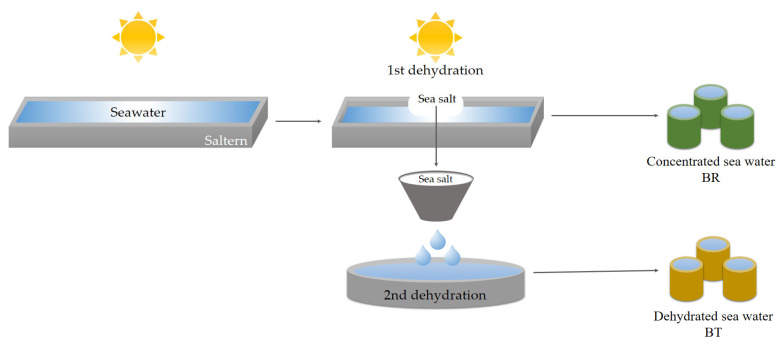
Processing method of seawater used as curing agent in this study.

Ocean and seawater consist of approximately 96.7% water and 3.3% salt, and the most positive ions, Na^+^, Mg^2+^, Ca^2+^, and K^+^ are concentrated [19]. There are two types of salt water that can be obtained from seawater: brine and bittern. Brine (BR; concentrated sea water) is seawater that remains after concentrating seawater to make salt, and bittern (BT; dehydrated brine) is seawater created during the dehydration process of sea salt (Figure 1). Bittern is generated in large quantities after passing through the process but is discarded [20]. It has already passed the safety test for food use in Korea and is used as tofu bittern, Kimchi agent, etc. [21,22,23]. The purpose of this study is to evaluate the added value of seawater as a curing agent, by differentiating the general and sensory characteristics of seawater-treated groups and the untreated, control group of meat.

## 2. Materials and Methods

### 2.1. Preparation of Wet-/Dry-Cured and Dry-Aged Bacon with Various Curing Agents

A total of 48 pork bellies from 24 animals were purchased from a local slaughterhouse and used 24 h after slaughter. The samples were prepared using 3 parts in the center after dividing the whole pork bellies into 5 equal parts of which a total of 144 samples were randomly assigned into 16 groups classified by combination (treatments 4 × periods 4 × repetitions 9). The seawater (brine and bittern) was purchased from Yeorumul (Incheon, Republic of Korea), at this time, the BR components were 5.89% sodium, 2.79% magnesium, 0.80% potassium, and 0.03% calcium, etc., and the BT components were 2.80% sodium, 17.90% magnesium chloride, 1.40% potassium, zinc, etc.).

Treatment groups were divided into—wet curing and dry aging (WA), dry curing and aging (DA), brine curing and dry aging (BR), and bittern curing and dry aging (BT). The curing agents were as follows: a salt-dissolving agent in water for wet curing, a solid salt agent for dry curing, brine (liquid) mixed with water for brine curing, and bittern (liquid) mixed with water for bittern curing. The salinity composition was 3.6% of liquid and 2.4% of salt itself based on 100 g of meat. For wet curing, the ratio of meat to prepared curing agent was 1:2. Additionally added contents were as follows: pepper 1.9%, brown sugar 1.1%, juniper berry 4.3%, nutmeg 1.9%, bay leaf 0.07%, and thyme 1.2%. Curing agents (WA, DA, BR, and BT) were weighed according to the weight of each belly and applied, and each sample was individually cured and vacuum-packed.

Curing was carried out for 7 days in a refrigerator at 4 °C and the samples were turned over every 24 h to ensure uniform salinity. Following this, the products were rinsed to completely remove agents, and dried at 15 °C and 60 RH% for 2 days. After drying, laurel leaves (0.67%), black pepper (4.35%), and chili pepper flakes (1.54%) were applied and aged for 3 weeks (0, 1, 2, and 3 weeks) in an aging chamber (storage conditions: 15 °C, RH 60%). At this time, drying was carried out in the same aging chamber, and the samples were dried carefully, hanging at regular intervals so that they did not touch each other.

### 2.2. Physicochemical and Microbial Composition Analysis

Aging yield (%) was calculated using the following formula by summing the 1-week salting process and 3-week drying process (total manufacturing period) compared to raw meat. Aging yield (%) = weight after aging (0–3 week)/raw meat × 100

Water activity (%) was measured at 25 °C using LabMaster-aw NEO (Novasina AG, Zurich, Switzerland). The water activity meter was calibrated at 0.064, 0.113, 0.328, 0.576, 0.753, 0.843, 0.973, and 1.000 aw levels, where 1.00 aw was considered as 100% RH.

The pH was homogenized using a homogenizer (HMZ-20DN, Poonglim Tech, Seongnam, Republic of Korea) after bringing the ratio of the sample to tertiary distilled water at 1:4. At this time, homogenization was carried out at a speed of 9000 rpm for 1 min, and the homogenized sample was measured using a pH meter (Model S220, Mettler-Tolede, Schwar-zenbach, Switzerland).

Salinity (%) was measured using Scionix ssm-1000 (Sxionix SSM, Deagu, Republic of Korea), and was calibrated with a 2% salinity correction solution. A homogeneous sample was measured after weighing it at a 1:4 ratio like in the pH evaluation, and it was expressed in terms of percentage.

Volatile basic nitrogen (VBN) was tested by citing the Sujiwo method [24] and calculated using the following formula: VBN (mg/100 g) = ((a − b) × F × 0.02 × 14.007)/(s) × 100, where, ‘a’ is the titration amount of the sample (mL), ‘b’ is the titration of blank (mL), ‘F’ is the standardization index of 0.02 N sulfuric acid, and ‘s’ is the sample weight (g) to be.

Thiobarbituric acid reactive substance (TBARS) was used after slightly modifying the distillation method of [25]. After first homogenizing by adding 50 mL of distilled water and 0.2 mL of 0.3% BHT to 10 g of the sample, 47.5 mL of distilled water, 2.5 mL of 4 N HCl, and 1 mL of antifoaming agent were added together and heated using a heating mantle. Then, 50 mL of the collected distillate was used in the experiment. A total of 5 mL of the collected distillate and 5 mL of TBA reagent were mixed in a ratio of 1:1 and heated at 100 °C for 30 min in a dark room. After cooling the reagent, it was calculated by measuring the absorbance at 538 nm. Then, using 1,1,3,3-tetra-ethoxypropane as a standard, the TBARS value was expressed as a concentration of malondialdehyde (MDA, mg/kg).

Microbial composition was confirmed to be *Staphylococcus* spp., *Lactobacillus* spp., yeast, mold, and general bacteria, using mannitol salt agar (MSA) medium, De Man, Rogosa and Sharpe agar (MRS agar) medium, potato dextrose agar (PDA) medium, and aerobic count plate (AC), respectively. An amount of 10 g of the sample and 2 counts of 0.1% buffer peptone water (BPW) were homogenized, and the filtrate was diluted according to the required dilution ratio and used. Thereafter, the MSA, MRS, and AC agar media in which the microorganisms were dispensed were cultured at 37 °C for 24 h, and the PDA medium was cultured at 25 °C for 48 h, followed by counting. The number of detected microorganisms was calculated and expressed as log CFU/g.

### 2.3. Electronic Nose

Using an electronic nose system (Heracles-II-e-nose, Alpha MOS, Toulouse, France), the volatile compounds was analyzed. After weighing 5 g of the sample in vials, they were sealed and kept refrigerated at 4 °C until the experiment was carried out. The samples were incubated in a controlled thermostatic agitator at 80 °C for 20 min. The samples were analyzed for flavor under the following conditions: headspace injection of 2.0 mL, injection speed of 200 μL/s, and injection temperature of 200 °C. MXT-5 and MXT-1701 were mounted, and the detector temperature was maintained at 260 °C. And then, the aroma pattern revealed the principal component analysis (PCA).

### 2.4. Fatty Acid Composition

For fatty acid composition analysis, 150 mL of a solvent (CM) in which chloroform and methanol were mixed at a ratio of 2:1 was added to 5 g of the sample, homogenized at 2500× *g* for 3 min, filtered, and 20 mL of 0.88% KCl was added. Then, sodium sulfate was added to the resultant lower layer after centrifugation at 3000 rpm for 10 min and filtered. The sample was concentrated between 560–565 °C using a rotary evaporator (N-1000, Eyela, Tokyo, Japan), the concentrated lipids were sealed with para film after injection of nitrogen gas, and stored frozen at −20 °C until methylation. To 200 μL of the sample, 1 mL of 0.5 N NaOH and 2 mL of 14% boron trifluoride were added, heated at 80 °C for 1 h and cooled for 10 min. Then, 5 mL of the sample was mixed with 2 mL of heptane, 2 mL of saturated NaCl solution was added, and left for 30 min. Then, 100 μL of the supernatant was taken and analyzed by gas chromatography (Agilent 7890, Agilent, Santa Clara, CA, USA). The column of the GC was HP-INNOWAX (30 m × 0.25 nm ID, 0.25 μm film) (Agilent 7890, Agilent, Santa Clara, CA, USA); the detector temperature was 260 °C; the injector temperature was 260 °C; the oven temperature was 100 °C for 2 min, 3 °C for a min, and 230 °C for 20 min; and nitrogen was used as the carrier gas.

### 2.5. Sensory Evaluation

Sensory evaluation was conducted in accordance with the guidelines of the Institutional Bioethics Committee of Kongju University (KNU_IRB_2021-54). Thirteen trained panelists were recruited, and they performed sensory evaluation in separate locations on the same day. Trained panelists are members who already have previous meat product evaluation experience. In addition, before carrying out the sensory evaluation of this study, they trained on the expression of taste, flavor, and texture senses more than 5 times within 10 days. Before performing the sensory evaluation, the panelists received additional training by referring to the sensory evaluation table of [26]. The sensory evaluation was conducted in one session. Bacon samples, which were dry aged with different curing agents, were sliced into 0.2 mm and served on a white plate. Sensory evaluation was carried out within 1 h, and each sample was divided into taste, flavor, and texture and tested by tasting (for example, taste after tasting, flavor after tasting, and texture after tasting). Each panelist evaluated a randomly selected sample according to a standard 9-point scale and was asked to give a high score to the ‘most preferred’ sample among the presented samples. The sensory evaluation included taste, flavor, and texture as salty/sour/greasy/overall, milky/cheesy/rancid/overall, and juicy/gummy/overall, respectively (excerpt from Reference [26]).

### 2.6. Statistical Analysis

Two general linear models were used to analyze the relationships of the treatment type (WA, DA, BR, and BT) and the production period (0, 1, 2, and 3 weeks) with measured traits except for volatile compound traits. For the traits that were evaluated once from the final products at 3 weeks, such as physicochemical properties, fatty acid composition, and sensory evaluation, the treatment type was fitted as a fixed effect in the model for a one-way analysis of variance. The model for microbial composition, which was measured each week, included the treatment type and the production period as fixed effects with their interaction effect. Then, the results were expressed as mean values and standard deviations; the significant differences between the mean values were determined by Duncan’s multiple range test (*p* < 0.05) except for the electronic nose value. The principal component analysis for volatile compounds was conducted by the Alpha soft program (Alpha MOS, Toulouse, France) using the electronic nose sensitivity values of the main volatile compounds.

## 3. Results and Discussion

### 3.1. Physicochemical Properties

Table 1 shows the results of the analysis on the physicochemical characteristics of dry-aged bacon that has been cured with different curing agents. Water activity and pH were significantly higher in BT than in other treatments (*p* < 0.05). It was confirmed that the water activity decreased as it approached the isoelectric point (IP) of meat, 5.2–5.4. Since high water activity can be utilized as a factor for the growth of pathogenic microorganisms such as *Listeria monocytogenes*, it is important to suppress it [27]. On the other hand, since high water activity has a positive effect on texture and tenderness in meat, packaging methods such as hydrostatic pressure processing can be applied to improve the food stability [28]. Low pH often results in a sour taste, this taste caused a low consumption rate. Salinity was higher in DA than WA, BR, and BT (*p* < 0.05). In the case of dry-curing process, since the difference in concentration between the inside and outside of the muscle was extreme, it led to more dehydration than wet curing, which was thought to have an effect on Aw [29]. However, high salt content caused low preference, so it is important to maintain low salinity and good taste. Dry yield did not show a significant difference among the treatment groups (*p* > 0.05).

VBN and TBARS of DA showed significantly higher values than those in other treatments (*p* < 0.05). Decomposition of proteins and rancidity of fats occurred most frequently during the curing period, and these were thought to be suppressed in the wet-curing processes of WA, BR, and BT, because access to oxygen was restricted due to immersion. Chaijan [30] reported that NaCl promotes lipid oxidation in the muscle tissue. Since the dry-cured ham prepared in this study did not exceed the VBN standard value applicable to fresh meat in Korea, 20 mg/100 g, it seems that all treatments in this study meet the regulations.

### 3.2. Microbial Composition

In fermented and dried meat products, fat oxidation, carbohydrate decomposition, and protein degradation occur due to microorganisms, and the microbial reaction is different depending on the type of food. Accordingly, the microbial composition of dry-aged bacon cured by using different curing agents was analyzed (Table 2). Aerobic bacteria showed significant differences in WA and DA compared to BT at week 1 (*p* < 0.05), but there was no difference thereafter. The aerobic bacteria by storage period increased and then decreased in WA, and those in BR were significantly lower at week 0 compared to other weeks (*p* < 0.05). The produced flavor was reported to be different because the decomposed and produced compounds are different depending on the strain of microorganisms growing in food [31]. Montel et al. [32] reported that the amount of lactic acid affects the sour taste.

In this study, *Staphylococcus* and *Lactobacillus* spp. were significantly higher in BR than DA at 2 weeks (*p* < 0.05), and *Staphylococcus* spp. were significantly higher in BT than WA and DA at 3 weeks (*p* < 0.05). *Lactobacillus* spp. showed a decreasing trend after increasing up to 2 weeks in all treatment groups, and *Staphylococcus* spp. showed an increasing trend. *Lactobacillus* spp. mainly occurring in fermented and aged meat products include *L. sakei* and *L. plantarum*, which are known to decompose proteins to produce free amino acids and thereby affect flavor [33]. On the other hand, since these microorganisms are mainly active at high temperatures and pHs, it is thought that their amount decreased with the increase of the storage period [34]. *Staphylococcus* spp. In meat include *S. xylosus*, *S. carnosus*, and *S. warneri*, and these are known to be involved in flavor expression by producing free fatty acids. Therefore, it was predicted that BR, rich in compounds produced by *Lactobacillus* spp., and BT, rich in compounds produced by *Staphylococcus* spp., would have a flavor different from that of WA and DA. According to Wang et al. [35], the free amino acid produced by *Lactobacillus* has a positive correlation as 2-nonenal, 2-undecenal, 1-octen-3-ol, 2,4-dimethylpent-1-en-3-ol, and malonamic acid. The free fatty acid produced by *Staphylococcus* has a positive correlation as benzoic acid, 2-[(trimethylsilyl)oxy]-, trimethylsilyl ester and (Z)-hept-2-enal. Thus, the microbial composition can enhance the unique dry-cured bacon flavor.

On the other hand, there was a significant difference in the numbers of mold and yeast, they were the highest in WA and lowest in DA (*p* < 0.05). BR and BT showed significantly higher values of mold than WA and DA at week 0 (*p* < 0.05), but at week 3, WA showed significantly higher values than other treatment groups (*p* < 0.05). *Debaryomyces hansenii* and *Penicillium chrysogenum* are yeasts and molds which decompose proteins and are mainly found in fermented meat products [36]. *P. chrysogenum* is known to play a role in softening firmness [37]. The dry-aged ham mold showed an increasing trend with the storage period in all treatment groups. It was therefore thought that different flavors would be expressed when amounts of molds and yeasts were significantly higher.

### 3.3. Electronic Nose

**Figure 2:**
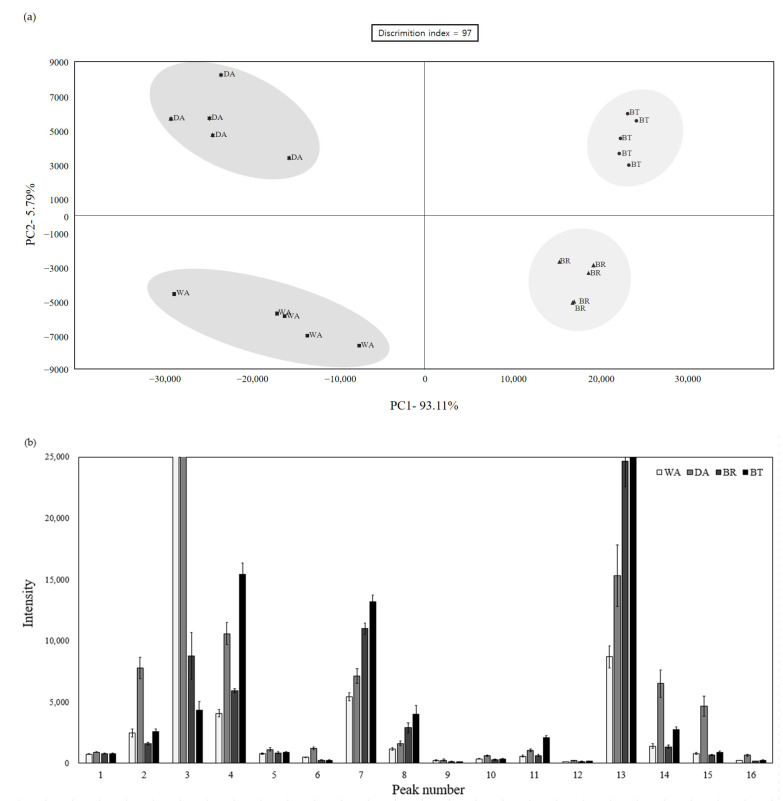
(**a**) Principal components analysis for dry-aged bacon ham with various curing agents. (**b**) Predicted volatile compounds in bacon ham cured with various curing agents. WA: wet curing and dry aging; DA: dry curing and dry aging; BR: wet curing with brine and dry aging; BT: wet curing with bittern and dry aging. Peaks are presented in order of elution: 1–3: acetaldehyde; 4: 2-propanol; 5: 2-methyl propanal; 6: 1-propanol; 7: butane-2,3-done; 8: butane-2-one; 9: acetic acid; 10: 1-hydroxy-2-propanone; 11: n-butanol; 12: propyl acetate (fermented); 13: pentanal; 14: 3-methyl 1 butanol; 15: 2-hexanol; and 16: butanoic acid.

The electronic nose can discriminate mixtures and emissions of volatile organic compounds, allowing their rapid identification [38]. Since the electronic nose can easily analyze the difference between completely different treatments, it was used in this study to differentiate bacon samples dry-aged with different types of curing agents (Figure 2). Figure 2a shows the principal components, and the four treatment groups were perfectly distinguished based on the 0 point of the x and y axes. The *x*-axis shows a high reliability of 92.34, and based on the *x*-axis, it is predicted as the difference between sea salt and seawater. Figure 2b shows the intensity of the predicated volatile compounds generated from the sample, and the peaks found at 10–60 s during flavor capture were analyzed. Peaks 1, 6, 9, and 10 represent acetaldehyde, 1-propanol, acetic acid, and 1-hydroxy 2-propanone, respectively; these compounds showed higher strength in WA- and DA-cured groups than in BR and BT groups. Among them, acetaldehyde, 1-propanol, and acetic acid are classified as primary aroma volatiles, and are compounds found in the early stages of aging.

They are distinguished by their free aroma, precursors of varietal origin (non-volatile or odorless precursors and odorous volatile compounds) and substances generated from precursors; it is known that these are expanded to secondary and tertiary aromas through fermentation and aging [39]. Therefore, it was predicted that the WA and DA in which these compounds appear will show a strong flavor of meat itself. Peaks 7, 8, and 13 were measured to be high in both BR and BT salted with seawater, and peaks 4, 7, 8, 11, 13, and 14 showed higher strength in BT than in other treatments. Peaks 4, 7, 8, 11, 13, and 14 represent a compound of 2-propanol, butane-2,3-dione, butane-2-one, n-butanol, pentanal, and 3-methyl 1-butanol; most of which are known to appear in the aging of fat, resulting in butter and cheese flavors [3]. Methyl or butane compounds have been reported as flavor precursors produced by microbial metabolism in the final stage of dry aging [40]. Pentanal is reported to be produced following lipid oxidation of linoleic, linolenic, and arachidonic fatty acids [8]; in our experiment, this compound showed high strength in BT. Therefore, it can be predicted that dry-aged bacon cured with BT would have a richer savory flavor compared to other treatments.

### 3.4. Fatty Acid Composition

The fat in meat is decomposed by various lipases and micro-organisms, and by mechanisms during the fermentation and aging period; it is decomposed into distinct fatty acids through multiple pathways to generate various flavors [41]. Table 3 shows the fatty acid composition of dry-cured bacon with different curing agents. BT showed a significantly higher value of gamma linolenic acid (GLA) than did other treatments (*p* < 0.05), and a strong significant difference between the treatments was observed. BT also showed a higher value of eicosapentaenoic acid (EPA) than did other treatments, and there was no significant difference with the DA treatment (*p* > 0.05). Hexanal and 2,4-decadienal, the primary oxides of GLA, have been reported to have a fried or greasy odor [42]. Aquilani et al. [43] reported that when EPA and DHA fatty acids were added to a pork burger, better sensory evaluation results were obtained. In addition, GLA and EPA belong to polyunsaturated fatty acids (PUFAs), and since they are not synthesized in the human body they need to be ingested in appropriate amounts [44]. PUFAs, n6, and n3 showed significantly higher values in the BT treatment group (*p* < 0.05). Canalis et al. [45] reported that PUFAs increased the antioxidant capacity of meat fat, suggesting that the nutritional value of the BT group, which contained a high volume of this component, was higher than that of other groups. Chen et al. [46] reported that different curing methods promoted various kinds of lipolysis, producing other free fatty acids, which may affect flavor. Overall, since BT yields a higher fatty acid composition (especially, significantly higher PUFAs) compared to other treatments, it produces tender [47] and has the potential to produce higher oily meat flavors.

### 3.5. Sensory Evaluation

Table 4 shows the sensory evaluation results of bacon dry-aged with different curing agents. For sensory evaluation, salty, sour, greasy, and overall parameters expressing taste; milky, cheesy, rancid, and overall, indicating flavor; and juicy, gummy, and overall indicating texture; were analyzed. There was no significant difference in taste between the treatments. In terms of flavor, the groups showed a difference except for the ‘rancid’ parameter, and BT generally showed the highest flavor. In particular, cheese flavor was significantly higher in BT than in other treatments (*p* < 0.05). It was attributed to compounds such as butane-2,3-dione, butane-2-one, and n-butanol. Butane-2,3-dione is a major aldehyde generated from the antioxidants of meat fat and is known to increase palatability [48]. In addition, it is thought that BT contains a high level of linoleic acid, which is oxidized during the aging process to produce a ripening effect, resulting in the same palatable flavor [49]. In terms of texture, BT showed a higher overall value than other treatments, and in the overall evaluation, it showed a significantly higher value than WA (*p* < 0.05). Dry curing is generally reported to obtain a higher level of acceptance compared to wet curing [6]; however, in terms of sensory evaluation according to flavor, BT showed more favorable results than other treatments. Accordingly, BT is considered to have new market potential as a curing agent.

## 4. Conclusions

The purpose of this study was to analyze the quality of dry-aged bacon aged using seawater (brine and bittern), which contains a large amount of minerals but is classified as a by-product. The seawater used in this experiment was processed and used for curing food. VBN, an indicator of the shelf life of meat, was lower in the seawater treatment groups (BR, BT) than in the control group. TBARS was higher in DA than other treatment groups, thus indicating a difference (wet cured, dry cured) according to the curing method. *Lactobacillus* spp., which affects protein degradation, and *Staphylococcus* spp., which affects lipolysis, showed the highest values in BR and BT, respectively, and expressed different flavors from those in the commonly used wet- and dry-cured methods. As for the electronic nose, since all treatments are perfectly divided into quadrants, in each quadrant, it is judged that there will be substances with different flavors. Differences in volatile compounds were confirmed between WA and DA treated with sea salt and BR and BT treated with seawater. It was confirmed that 2-propanol, butane-2,3-done, and pentanal expressing an aged flavor was the strongest in the BT group. In addition, the BT group was confirmed to contain a large amount of PUFAs compared to other treatments, which are known for health benefits; the resultant enhanced flavor profile was also identified by the electronic nose. The sensory evaluation was performed for taste, flavor, and texture individually, and flavor was shown to be more influenced than the sense of taste. Overall, BT was found to have a rich milky and cheesy flavor. Because bittern showed mostly equal or superior results compared to the control, it is thought to hold significant potential as a curing agent. In addition, this study also provided value addition to wasted seawater.

## Figures and Tables

**Table 1:** Physicochemical properties in dry-aged bacon ham with various curing agents aged 3 weeks.

Table	Treatments ^1^				*p*-Value
	WA	DA	BR	BT	
pH	5.65 ± 0.05 ^bc^	5.68 ± 0.01 ^b^	5.61 ± 0.05 ^c^	5.92 ± 0.08 ^a^	***
Water activity (%)	93.16 ± 0.55 ^b^	92.41 ± 0.06 ^c^	92.51 ± 0.04 ^c^	93.83 ± 0.16 ^a^	**
Salinity (%)	1.41 ± 0.18 ^b^	1.61 ± 0.27 ^a^	1.35 ± 0.11 ^b^	1.38 ± 0.13 ^b^	*
Aging yield (%)	75.66 ± 1.66 ^NS^	74.95 ± 1.30	75.87 ± 1.56	75.36 ± 1.12	^NS^
VBN (mg/100 g)	4.01 ± 0.86 ^b^	5.08 ± 1.38 ^a^	2.89 ± 1.11 ^c^	3.45 ± 0.25 ^bc^	***
TBARS (MDA/g)	0.96 ± 0.07 ^b^	2.27 ± 0.24 ^a^	0.84 ± 0.07 ^b^	0.81 ± 0.04 ^b^	***

All values are mean ± standard deviations. ^1^ WA, wet curing and dry aging; DA, dry curing and dry aging; BR, wet curing with brine and dry aging; BT, wet curing with bittern and dry aging. * *p* < 0.05; ** p < 0.01; *** p < 0.001; ^NS^ non-significant. ^a–c^ Mean in the same row with different letters are significantly different (*p* < 0.05).

**Table 2:** Microbial population in dry-aged bacon ham with various curing agents during the aging period.

Table ^1^	Treatments ^2^	Aging Period (Week)				*p*-Value ^3^
		0	1	2	3	
AC	WA	6.78 ± 0.88 ^b^	7.41 ±0.36 ^Aa^	7.46 ± 0.36 ^a^	7.26 ± 0.42 ^ab^	T ** P *** T × P ^NS^
	DA	6.75 ± 0.93	6.91 ± 0.48 ^AB^	7.17 ± 0.48	7.06 ± 0.48	
	BR	6.59 ±0.43 ^b^	7.16 ± 0.60 ^ABa^	7.37 ± 0.37 ^a^	7.26 ± 0.50 ^a^	
	BT	6.53 ± 0.30	6.70 ± 0.92 ^B^	7.02 ± 0.28	6.81 ± 0.51	
MRS	WA	6.01 ± 0.34 ^b^	6.29 ± 0.33 ^b^	6.87 ± 0.58 ^ABa^	6.19 ± 0.89 ^b^	T ^NS^ P *** T × P ^NS^
	DA	5.92 ± 0.33 ^b^	6.25 ± 0.46 ^a^	6.42 ± 0.12 ^Ba^	6.20 ± 0.26 ^ab^	
	BR	5.80 ± 0.18 ^c^	6.42 ± 0.46 ^b^	7.10 ± 0.74 ^Aa^	6.30 ± 0.72 ^bc^	
	BT	5.87 ± 0.51 ^c^	6.15 ± 0.35 ^bc^	6.62 ± 0.32 ^ABa^	6.35 ± 0.04 ^ab^	
MSA	WA	4.59 ± 0.26 ^b^	5.03 ± 0.89 ^b^	5.24 ± 0.75 ^ab^	5.72 ± 0.34 ^Ba^	T ^NS^ P *** T × P ^NS^
	DA	4.64 ± 1.22 ^b^	4.93 ± 0.80 ^b^	5.18 ± 0.81 ^ab^	5.83 ± 0.49 ^Ba^	
	BR	4.03 ± 0.35 ^c^	4.64 ± 1.25 ^bc^	5.04 ± 1.13 ^b^	6.14 ± 0.58 ^ABa^	
	BT	4.26 ± 0.59 ^b^	4.72 ± 1.17 ^b^	5.10 ± 0.55 ^b^	6.52 ± 0.37 ^Aa^	
PDA	WA	4.34 ± 0.69 ^Bc^	4.99 ± 0.65 ^b^	5.96 ± 0.54 ^a^	6.15 ± 0.24 ^Aa^	T ^NS^ P *** T × P ^NS^
	DA	4.29 ± 0.55 ^Bb^	5.19 ± 0.98 ^a^	5.50 ± 1.02 ^a^	5.66 ± 0.26 ^Ca^	
	BR	5.16 ± 0.32 ^Ac^	5.37 ± 0.60 ^bc^	5.68 ± 0.66 ^ab^	5.91 ± 0.16 ^Ba^	
	BT	4.91 ± 0.31 ^Ab^	5.15 ± 1.22 ^ab^	5.60 ± 0.30 ^ab^	5.85 ± 0.31 ^BCa^	

All values are mean ± standard deviations. ^1^ AC, aerobic count plate; MRS, *Lactobacillus* spp. count plate; MSA, *Staphylococcus* spp. count plate PDA, yeast, and mold count plate. ^2^ WA, wet curing and dry aging; DA, dry curing and dry aging; BR, wet curing with brine and dry aging; BT, wet curing with bittern and dry aging. ^3^ T, treatment; P, aging period; T × P, treatment × aging period. ** *p* < 0.01; *** *p* < 0.001; ^NS^ non-significant. ^a–c^ Mean in the same row with different letters are significantly different (*p* < 0.05). ^A–C^ Mean in the same column with different letters are significantly different (*p* < 0.05).

**Table 3:** Fatty acid (% of total fatty acids) in dry-aged bacon ham with various curing agents aged 3 weeks.

Traits ^1^	Treatments ^2^				*p*-Value
	WA	DA	BR	BT	
C14:0	1.400 ± 0.02 ^NS^	1.458 ± 0.07	1.459 ± 0.02	1.379 ± 0.04	^NS^
C16:0	24.937 ± 0.22 ^NS^	26.541 ± 1.58	25.040 ± 1.10	24.987 ± 1.70	^NS^
C16:1n7	1.482 ± 0.01 ^NS^	1.423 ± 0.14	0.935 ± 0.12	1.466 ± 0.04	^NS^
C18:0	12.434 ± 0.68 ^NS^	10.691 ± 1.79	13.544 ± 0.71	10.155 ± 1.36	^NS^
C18:1n9	42.800 ± 0.43 ^NS^	41.338 ± 1.77	43.340 ± 1.02	42.598 ± 0.82	^NS^
C18:1n7	0.102 ± 0.04 ^NS^	0.127 ± 0.02	0.104 ± 0.05	0.120 ± 0.05	^NS^
C18:2n6	14.696 ± 0.14 ^NS^	15.952 ± 1.03	13.502 ± 0.23	16.739 ± 0.97	^NS^
C18:3n6	0.017 ± 0.01 ^b^	0.018 ± 0.01 ^b^	0.023 ± 0.00 ^b^	0.029 ± 0.01 ^a^	***
C18:3n3	1.169 ± 0.06 ^NS^	1.330 ± 0.18	0.991 ± 0.04	1.397 ± 0.16	^NS^
C20:1n9	0.588 ± 0.08 ^NS^	0.704 ± 0.09	0.649 ± 0.09	0.632 ±0.24	^NS^
C20:4n6	0.267 ± 0.01 ^NS^	0.306 ± 0.01	0.300 ± 0.01	0.362 ± 0.02	^NS^
C20:5n3	0.013 ± 0.01 ^b^	0.015 ± 0.01 ^ab^	0.011 ± 0.01 ^b^	0.019 ± 0.02 ^a^	*
C22:4n6	0.066 ± 0.01 ^NS^	0.068 ± 0.01	0.077 ± 0.01	0.083 ± 0.01	^NS^
C22:6n3	0.029 ± 0.01 ^NS^	0.030 ± 0.01	0.025 ± 0.01	0.036 ± 0.01	^NS^
SFA	38.770 ± 0.13 ^NS^	38.691 ± 1.01	40.043 ± 1.06	36.521 ± 0.62	^NS^
UFA	61.230 ± 0.48 ^NS^	61.309 ± 1.18	59.957 ± 1.83	63.479 ± 0.34	^NS^
MUFA	44.973 ± 0.56 ^NS^	43.591 ± 1.97	45.027 ± 1.00	44.815 ± 0.49	^NS^
PUFA	16.257 ± 0.08 ^bc^	17.718 ± 1.21 ^ab^	14.929 ± 0.18 ^c^	18.664 ± 1.11 ^a^	*
UFA/SFA	1.580 ± 0.03 ^NS^	1.597 ± 0.22	1.500 ± 0.11	1.739 ± 0.05	^NS^
MUFA/SFA	1.160 ± 0.03 ^NS^	1.135 ± 0.15	1.127 ± 0.10	1.227 ± 0.01	^NS^
PUFA/SFA	0.419 ± 0.01 ^ab^	0.462 ± 0.07 ^ab^	0.373 ± 0.01 ^b^	0.511 ± 0.04 ^a^	^NS^
n6	15.046 ± 0.14 ^bc^	16.344 ± 1.03 ^ab^	13.902 ± 0.22 ^b^	17.211 ± 0.95 ^a^	*
n3	1.211 ± 0.06 ^ab^	1.374 ± 0.18 ^ab^	1.028 ±0.04 ^b^	1.452 ± 0.16 ^a^	^NS^
n6/n3	12.439 ± 0.73 ^NS^	11.959 ± 0.77	13.541 ± 0.77	11.886 ± 0.63	^NS^

^1^ SFA, saturated fatty acids = C12:0 + C14:0 + C15:0 + C16:0 + C17:0 + C18:0; UFA, unsaturated fatty acids = C14:1 + C15:1 + C16:1 + C17:1 + C18:1 n − 9 + C18:1 n − 9 trans + C18:2 n − 6 + C18:3 n − 3 + C20:4 n − 6, C20:5 n − 3 + C22:5 n − 3 + C22:6 n − 3; MUFA, monounsaturated fatty acids = C14:1 + C15:1 + C16:1 + C17:1 + C18:1 n − 9 + C18:1 n − 9 trans; PUFA, polyunsaturated fatty acid, PUFA n − 3 = C18:3 n − 3 + C20:5 n − 3 + C22:5 n − 3 + C22:6 n − 3. PUFA n − 6 = C18:2 n − 6 + C20:4 n − 6. ^2^ WA, wet curing and dry aging; DA, dry curing and dry aging; BR, wet curing with brine and dry aging; BT, wet curing with bittern and dry aging. * *p* < 0.05; *** *p* < 0.001; NS, non-significant. ^a–c^ Mean in the same row with different letters are significantly different (*p* < 0.05).

**Table 4:** Sensory evaluation in dry-aged bacon ham with various curing agents aged 3 weeks.

Traits		Treatments ^1^				*p*-Value
		WA	DA	BR	BT	
Taste	Salty	4.38 ± 0.95	4.82 ± 0.88	4.05 ± 0.93	5.09 ± 1.26	^NS^
	Sour	4.56 ± 1.07	5.20 ± 0.88	4.27 ± 1.12	5.36 ± 1.03	^NS^
	Greasy	4.91 ± 0.71	5.14 ± 0.81	4.82 ± 0.94	5.27 ± 0.99	^NS^
	Overall	5.36 ± 1.42	5.50 ± 0.61	5.45 ± 0.82	5.73 ± 1.42	^NS^
Flavor	Milky	4.25 ± 0.89 ^b^	5.17 ± 1.33 ^ab^	4.92 ± 1.02 ^ab^	6.00 ± 0.89 ^a^	*
	Cheesy	4.83 ± 0.75 ^b^	5.30 ± 1.06 ^b^	4.86 ± 1.07 ^b^	6.14 ± 0.69 ^a^	*
	Rancid	5.63 ± 1.30	5.50 ± 1.07	5.38 ± 1.19	5.89 ± 0.93	^NS^
	Overall	4.83 ± 1.22 ^b^	5.50 ± 0.79 ^ab^	5.06 ± 0.81 ^b^	6.09 ± 0.83 ^a^	*
Texture	Juicy	4.82 ± 1.17 ^b^	5.91 ± 0.94 ^a^	5.82 ± 0.75 ^a^	5.94 ± 0.81 ^a^	*
	Gummy	5.00 ± 1.73 ^b^	5.78 ± 0.83 ^ab^	5.55 ± 0.69 ^ab^	6.10 ± 0.74 ^a^	^NS^
	Overall	4.86 ± 1.52 ^b^	5.77 ± 1.09 ^ab^	5.50 ± 0.53 ^ab^	6.09 ± 0.83 ^a^	^NS^

^1^ WA: wet curing and dry aging; DA: dry curing and dry aging; BR: wet curing with brine and dry aging; BT: wet curing with bittern and dry aging. * *p* < 0.05; NS, non-significant. ^a,b^ Mean in the same row with different letters are significantly different (*p* < 0.05). 1 = “strongly disagree” to 5 = “strongly agree”.

## Data Availability

Data is contained within the article.
